# Increased growth rate and amikacin resistance of *Salmonella enteritidis *after one‐month spaceflight on China’s Shenzhou‐11 spacecraft

**DOI:** 10.1002/mbo3.833

**Published:** 2019-03-25

**Authors:** Bin Zhang, Po Bai, Xian Zhao, Yi Yu, Xuelin Zhang, Diangeng Li, Changting Liu

**Affiliations:** ^1^ Nankai University School of Medicine Tianjin China; ^2^ Respiratory Diseases Department The Second Medical Center of Chinese PLA General Hospital Beijing China; ^3^ Respiratory Diseases Department PLA Rocket Force Characteristic Medical Center Beijing China

**Keywords:** genome, phenotype, proteome, *Salmonella enteritidis*, spaceflight, transcriptome

## Abstract

China launched the Tiangong‐2 space laboratory in 2016 and will eventually build a basic space station by the early 2020s. These spaceflight missions require astronauts to stay on the space station for more than 6 months, and they inevitably carry microbes into the space environment. It is known that the space environment affects microbial behavior, including growth rate, biofilm formation, virulence, drug resistance, and metabolism. However, the mechanisms of these alternations have not been fully elucidated. Therefore, it is beneficial to monitor microorganisms for preventing infections among astronauts in a space environment. *Salmonella enteritidis *is a Gram‐negative bacterial pathogen that commonly causes acute gastroenteritis in humans. In this study, to better understand the effects of the space environment on *S. enteritidis, *a *S. enteritidis *strain was taken into space by the Shenzhou‐11 spacecraft from 17 October 2016 to 18 November 2016, and a ground simulation with similar temperature conditions was simultaneously performed as a control. It was found that the flight strain displayed an increased growth rate, enhanced amikacin resistance, and some metabolism alterations compared with the ground strain. Enrichment analysis of proteome revealed that the increased growth rate might be associated with differentially expressed proteins involved in transmembrane transport and energy production and conversion assembly. A combined transcriptome and proteome analysis showed that the amikacin resistance was due to the downregulation of the *oppA* gene and oligopeptide transporter protein OppA. In conclusion, this study is the first systematic analysis of the phenotypic, genomic, transcriptomic, and proteomic variations in *S. enteritidis* during spaceflight and will provide beneficial insights for future studies on space microbiology.

## 1 INTRODUCTION

With the rapid development of manned‐spaceflight technology, space environment has become a new field of human activity. In space environment, although complex factors such as microgravity, cosmic radiation, and extreme temperature may impact the behavior of living organisms, many microbes have adapted to survive under these conditions. For instance, some microbial isolates were collected from astronauts and filter debris during orbital spaceflights on the International Space Station (ISS) (Checinska et al., 2015; Venkateswaran et al., 2014). It has been reported that the space environment can alter the microbial growth rate, virulence, and antibiotic susceptibility (Kim et al., 2013; Klaus & Howard, 2006; Mora et al., 2016; Rosenzweig, Ahmed, Eunson, & Chopra, 2014; Taylor, 2015; Urbaniak et al., 2018; Wilson et al., 2007). In addition, many studies have shown that the extreme environment of space plays an important role in dysregulation of the human immune system (Crucian et al., 2018; Kaur, Simons, Castro, Ott, & Pierson, 2005; Taylor, 2015). Animal experiments also indicated that splenic lymphocyte, monocyte/macrophage, and granulocyte counts were significantly reduced in mice during spaceflight compared with ground control mice (Baqai et al., 2009). These changes in the microbes and human body may increase the risk for unexpected infectious diseases, thus impairing the health of astronauts. It has been observed that spaceflight could compromise the balance between the human body and human microbiome, induce the immune dysregulation of astronauts, and finally increase the risk for microbial infections (Cervantes & Hong, 2016). Some recent studies indicated that space travel changed the human microbiota, which modulated the risks of various health conditions, and probiotic interventions might help to reduce the risks of infectious diseases as well as immune dysfunction during spaceflight (Douglas & Voorhies, 2017; Urbaniak & Reid, 2016). Therefore, monitoring changes in microorganisms in the space environment is beneficial for preventing and curing infectious diseases (Liu, 2017). However, research concerning how microbes respond differentially to space environmental conditions is scarce. Therefore, the elucidation of the underlying mechanisms of spaceflight effects is urgently needed for astronauts and the general population.

*Salmonella enteritidis* is a Gram‐negative bacterial pathogen that exists in the intestinal tract, skin, and feathers of living birds. *S. enteritidis* induces gastrointestinal and systemic diseases in human and animal hosts after the uptake of contaminated water and food such as poultry meat and eggs (Mirhosseini, Fooladi, Amani, & Sedighian, 2017; Tarabees, Elsayed, Shawish, Basiouni, & Shehata, 2017). The most commonly reported infectious disease caused by *S. enteritidis* is acute gastroenteritis (Muvhali, Smith, Rakgantso, & Keddy, 2017). This pathogen also causes systemic diseases such as bacteremia in humans, particularly in individuals with immune deficiency (Phoba et al., 2014). Notably, some *S. enteritidis *strains are highly resistant to antibiotics and pose a severe threat to global public health (Khumalo, Saidi, & Mbanga, 2014). The most traditional drug resistance mechanism of *S. enteritidis* is enzymatic detoxification of antibiotics (Radford et al., 2018). *S. enteritidis *to date has been discovered from the surface of stowage racks on the ISS, it may be carried into the space environment by human space activity and pose threats to the health of astronauts (Singh, Wood, Karouia, & Venkateswaran, 2018). Therefore, studying the impacts of spaceflight on *S. enteritidis *helps not only in evaluating the risk of infectious diseases among astronauts but also in detecting treatment targets against the bacteria. However, the impact of the space environment on *S. enteritidis* has not been determined yet, and no research has investigated whether and how space exposure changes growth rate, antibiotic susceptibility, and metabolism of *S. enteritidis*. In this study, we studied the phenotypic variations of *S. enteritidis* and used combined genomic, transcriptomic, and proteomic analyses to reveal the impacts of the extreme space environment on *S. enteritidis *during spaceflight on the Shenzhou‐11 spacecraft from 17 October 2016 to 18 November 2016.

## 2 MATERIALS AND METHODS

### 2.1 Bacterial strains and culture conditions

The original *S. enteritidis* strain was obtained from a stool sample of a patient with acute gastroenteritis at the Chinese PLA General Hospital. The original *S. enteritidis* strain (designated as SEO strain) was stored at −80°C and used as a reference. SEO was inoculated into two plastic containers filled with Luria–Bertani (LB) medium before the launch of Shenzhou‐11 spacecraft. The special plastic container was designed for this study as previously described (Su et al., 2014). The LB medium contained tryptone (10 g/L), yeast extract (5 g/L), NaCl (10 g/L), and agar powder (15 g/L), and the pH of the medium was adjusted to 7.0–7.2 (Guo et al., 2014). The strain (designated as SES strain) in one plastic container was stored in a liquid nitrogen transport tank and left the laboratory of the Chinese PLA General Hospital at 01:12 on 17 October 2016 (GMT +8). It was transported to Jiuquan Satellite Launch Center by military plane. Then, the sample in the plastic container was transferred to an onboard −80°C freezer and finally carried into the space environment by the Shenzhou‐11 spacecraft launched at 07:30 on 17 October 2016 (GMT +8). The Shenzhou‐11 spacecraft arrived at the Tiangong‐2 space station and successfully docked with the space station at 03:31 on October 19 (GMT +8). After docking, the sample in the plastic container was transferred from storage at −80°C to a 4°C refrigerator, thawed overnight, and grew at the cabin temperature. The Shenzhou‐11 spacecraft and Tiangong‐2 remained connected for 30 days at an approximate apogee distance of 393 km. Growth of the sample was terminated by transfer of the plastic container to the onboard −80°C freezer when the return capsule of the Shenzhou‐11 spacecraft completed the experimental task and left the Tiangong‐2 space station at 12:41 on November 17. The return capsule of the Shenzhou‐11 spacecraft landed at Siziwang Banne at 14:07 on November 18 (GMT +8). The microbiological sample was quickly removed into a liquid nitrogen transport tank and transported to Beijing by military plane. The sample arrived at the laboratory of the Chinese PLA General Hospital at 20:02 on November 18 (GMT +8). The strain (designated as SEG strain) in the other plastic container was cultured on the ground as a control experiment. This plastic container was simultaneously put into an incubator to simulate the temperature of the space environment. The temperature in the spacecraft was reported each hour and ranged from 19°C to 23°C (20.96 ± 1.30°C). Relevant temperature data were obtained by a temperature measuring device equipped in the cabin. After the spacecraft landed on Earth, all three strains were immediately grown on solid agar plates for further investigation.

### 2.2 Phenotypic analysis

#### 2.2.1 Growth rate assay

A bacterial growth curve was measured at 600 nm in a Bioscreen C system (Lab Systems, Finland). The strains were grown in LB liquid medium at 37°C overnight. After a 20 µl suspension, sample (10^6 ^CFU/ml) was inoculated into a 96‐well honeycomb microtiter plate, 350 µl fresh LB liquid medium was added into each well, and the microtiter plate was continuously shaken at the maximum amplitude for 24 hr. The growth curve was generated based on the optical density (OD) value at 600 nm. A well with only 370 µl LB liquid medium served as a blank control. This experiment was performed in three replicates.

#### 2.2.2 Antibiotic susceptibility test

Bacterial susceptibility to antibiotics was tested using the disk diffusion method. Ten antibiotics, including trimethoprim sulfamethoxazole (SXT, 25 µg), ciprofloxacin (CIP, 5 µg), piperacillin and tazobactam (TZP, 110 µg), cefoperazone and sulbactam (SCF, 105 µg), ampicillin (AMP, 10 µg), cefepime (FEP, 30 µg), amikacin (AK, 30 µg), levofloxacin (LEV, 5 µg), ceftazidime (CAZ, 30 µg) and meropenem (MEM, 10 µg), were used to test the sensitivity of the three strains. The entire surface of the LB agar plate was covered with bacterial inoculum (10^7^–10^8 ^CFU/ml), and then the antibiotic disks were placed on the surface of the plate after the plate was dried for 15 min. The diameter of the inhibition zone was measured to determine the antibiotic susceptibility of the bacteria in a standard specification following incubation for 18 hr at 37°C. This disk diffusion test was performed in triplicate.

#### 2.2.3 Carbon source utilization and chemical sensitivity assays

The Biolog GEN III MicroPlate (Biolog, CA) was used to study biochemical features of the three strains in 94 phenotypic tests, including 71 carbon source utilization and 23 chemical sensitivity assays. The strains were grown on the agar plate overnight at 37°C and added to the IF‐A inoculum (Biolog, CA). The concentration of bacterial suspension was adjusted to 10^8 ^CFU/ml by turbidimeter and then 0.1 ml of the suspension was inoculated into each well of the microplate. After incubation for 24 hr at 37°C, the microplate was measured using an automated Biolog microplate reader at OD 590 nm. This experiment was performed in triplicate.

### 2.3 Genome sequencing and annotation

#### 2.3.1 Genome sequencing and assembly

The bacterial DNA samples were obtained using the conventional phenol–chloroform extraction method (Javadi et al., 2014). After electrophoretic detection, qualified DNA samples were sheared into small fragments by Covaris S/E210, and then the overhangs of DNA fragments were converted into blunt ends using T4 DNA polymerase, Klenow fragment, and T4 Polynucleotide Kinase. An “A” base was added onto the 3’ end of the blunt phosphorylated DNA fragments, and adapters were ligated to the ends of fragments. After gel electrophoresis, the purified fragments were enriched and amplified by polymerase chain reaction (PCR). Finally, a 10 kb SMRT Bell library was constructed. The whole genome was sequenced using the PacBio platform according to the standard protocol, and polymerase reads were obtained. The raw reads were then filtered through the following steps: removing the polymerase reads with length <1 kb, removing the polymerase reads with low quality (<Q 0.8), removing adapter contamination, subtracting subreads from polymerase read, removing the subreads with length <1 kb and removing the subreads with low quality (<Q 0.8). Self‐correction was carried out using the software Falcon (Version 0.3.0), and corrected reads were acquired. Finally, the genome sequences were assembled using the software Celera Assembler (Version 8.3). In addition, to improve the accuracy of the genome sequences, GATK and SOAP tool packages were used to make single‐base corrections.

#### 2.3.2 Genome component prediction

Genome component prediction included the prediction of coding genes, noncoding RNAs interspersed and tandem repeat sequences, prophages and clustered regularly interspaced short palindromic repeat sequences (CRISPRs). The detailed steps were performed as follows: (a) the related coding genes were predicted by the software Glimmer; (b) transfer RNA (tRNA) genes, ribosome RNA (rRNA) genes, small nuclear RNA (snRNA) genes were recognized by tRNAscan‐SE software, RNAmmer software and Rfam database, respectively; (c) the tandem repeat annotation was retrieved by the Tandem Repeats Finder (Version 4.04), and the minisatellite DNA and microsatellite DNA were selected according to the number and length of repeat units; (d) the prophage regions were predicted by the software PHAST (Version 2013.03.20); and (e) CRISPR identification was predicted by CRISPRFinder (Version 0.4).

#### 2.3.3 Genome function analysis

Seven databases containing KEGG (Kyoto Encyclopedia of Genes and Genomes), COG (Clusters of Orthologous Groups), NR (Non‐Redundant Protein Database), GO (Gene Ontology), Swiss‐Prot, TrEMBL, and EggNOG were used for general gene function annotation. A whole‐genome blast alignment was analyzed by seven databases.

### 2.4 Comparative genomic analysis

#### 2.4.1 SNPs

The raw reads of each query sequence were identified by the alignment software MUMmer (Version 3.22) and SOAPaligner (Version 3.22). Low‐quality reads, including consecutive bases covered by fewer than five reads, were discarded. The clean reads were assembled by SOAPdenovo (Version 1.05). The variation sites of the query sequence were sought out and filtered to detect potential SNP sites. BLAT (Version 34) software was used to verify SNP sites, and the erroneous SNPs were removed. SNPs in repeat regions were predicted by BLAST (Version 2.2.2), TRF (Version 4.04) and Repeatmask (Version 3.2.9) software. The credible SNPs could be acquired by filtering SNPs located in repeat regions.

#### 2.4.2 Indel calling

The software SOAPdenovo (Version 1.05) was used to assemble the sequenced reads. To improve the reliability and accuracy, potential indels between the query genome and the reference genome were predicted using the software LASTZ (Version 1.01.50) with default parameters. The best alignment results were obtained and the preliminary indel results were obtained through a series of dispositions with axt correction, axtSort, and axtBest programs. The indels were verified by comparing the query genome to the surrounding region of the indels with the reference genome 150 bp upstream and 150 bp downstream by using BWA (Version 0.5.8). The indels that had reads absolutely mapped to the sites mentioned above in the reference genome were eliminated. The indels were retained if more than two reads were unmapped in the reference genome.

### 2.5 Transcriptome sequencing and comparative transcriptomic analysis

#### 2.5.1 Sequencing and filtering

Bacterial cells were collected after centrifugation at 8,000 g for 5 min at 4°C and mixed with 1.5 ml TRIzol reagent completely. The samples were centrifuged at 10,000*g* for 5 min at 4°C. The supernatant was added to 300 µl chloroform, shaken for 15 s, and centrifuged at 10,000*g* for 10 min at 4°C. Then, 900 µl supernatant was transferred into a tube containing 900 µl isopropanol and centrifuged at 13,600*g* for 20 min at 4°C. After removing the supernatant, the sample was mixed with 1 ml ethanol and centrifuged at 12,000*g* for 3 min at 4°C. Subsequently, the supernatant was removed, and the sample was centrifuged at 12,000*g* for 20 s at 4°C. After removing the residual liquid and air‐drying, the RNA pellet was dissolved in RNase‐free water. Total RNA was extracted by TIANGEN RNAprep pure Kit (Beijing, China) following the manufacturer's instructions. The mRNAs containing poly‐A were purified using poly‐T oligo‐attached magnetic beads. The purified mRNAs were fragmented into small pieces (~200 bp) using divalent cations. The RNA fragments were used to generate first strand cDNA by reverse transcriptase and random primers. Then, the second strand cDNA was created using DNA Polymerase I and RNase H. The cDNA fragments were enriched with PCR amplification and quantified by Qubit 2.0. Finally, the cDNA libraries were constructed. The libraries were sequenced using BGISEQ‐500. Raw reads were filtered using the software SOAPnuke (Version 1.5.2), and clean reads were acquired by removing adapter reads, poly‐N reads, and reads with 40 bp of low quality (<Q 15) base numbers.

#### 2.5.2 Gene expression value analysis

The RNAseq reads were mapped to the reference genome using the software HISAT (Version 2.0.4) (Kim, Langmead, & Salzberg, 2015) and Bowtie2 (Version 2.2.5) (Langmead & Salzberg, 2012). The gene expression level of each sample was analyzed using the software RESM (Version 1.2.12), and FPKM (expected number of fragments per kilobase of transcript sequence per million base pairs sequenced) was provided to count the gene expression value statistics. Cluster analysis of gene expression was performed using the software Cluster (Version 3.0), and the results of cluster analysis were displayed with JavaTreeView.

#### 2.5.3 Differential gene expression analysis

Differential gene expression was analyzed using the DESeq method, and genes with a fold change >2 and *p*‐value <0.05 in two different samples were defined as differentially expressed genes (DEGs). Then, the identified DEGs were enriched and clustered according to KEGG functional annotation.

### 2.6 Proteomic analysis

#### 2.6.1 The MS/MS raw data acquisition

The samples in liquid LB medium were collected by centrifugation at 25,000*g* for 15 min at 4°C. After discarding the supernatant, the samples were mixed with 1 ml PBS completely and centrifuged at 25,000*g* for 15 min at 4°C. The supernatant was removed, and lysis buffer was added to the samples. The suspension was sonicated for 1 min and centrifuged at 25,000*g* for 15 min at 4°C. After adding 10 mM DDT, the supernatant was incubated for 1 hr at 56°C. Subsequently, the samples were incubated with 55 mM IAM (iodoacetamide) for 45 min in the dark room for alkylation. Finally, the supernatant containing proteins was extracted after centrifugation at 25,000*g* for 15 min at 4°C. The proteins were quantified by Bradford and subsequently confirmed using SDS‐PAGE. After trypsin digestion, the peptides were labeled using iTRAQ reagents and fractionated using an LC‐20AB HPLC Pump system (Shimadzu, Kyoto, Japan) coupled with a high‐pH RP column. The processed peptides were separated using an LC‐20AD nano‐HPLC instrument (Shimadzu, Kyoto, Japan) with an autosampler. Then, the peptides purified from nanoHPLC were subjected to the tandem mass spectrometry Q EXACTIVE (Thermo Fisher Scientific, San Jose, CA) for DDA (data‐dependent acquisition) detection by nanoelectrospray ionization.

#### 2.6.2 Protein identification

The raw MS/MS data were converted into MGF format by the Thermo Scientific tool Proteome Discoverer, and the exported MGF files were searched against the selected genome annotation database using the software Mascot (Version 2.3.02) (Brosch, Yu, Hubbard, & Choudhary, 2009). In addition, proteins that were eventually identified must contain at least one unique peptide.

#### 2.6.3 iTRAQ protein quantification and functional annotation

Protein quantification was carried out using the isobaric tags for the relative and absolute quantitation (iTRAQ) method. All the proteins with a false discovery rate (FDR) less than 1% were selected to proceed using the software iQuant (Wen et al., 2014). The proteins with a fold change >1.2 and *p*‐value <0.05 in two different samples were defined as differentially expressed proteins (DEPs). The cluster analysis of protein expression patterns in different sample groups was performed using Euclidean distance and a hierarchical algorithm. Further analysis based on DEPs, including KEGG pathway enrichment analysis and COG function annotation, was performed.

## 3 RESULTS

### 3.1 Phenotypic analysis

**Figure 1:**
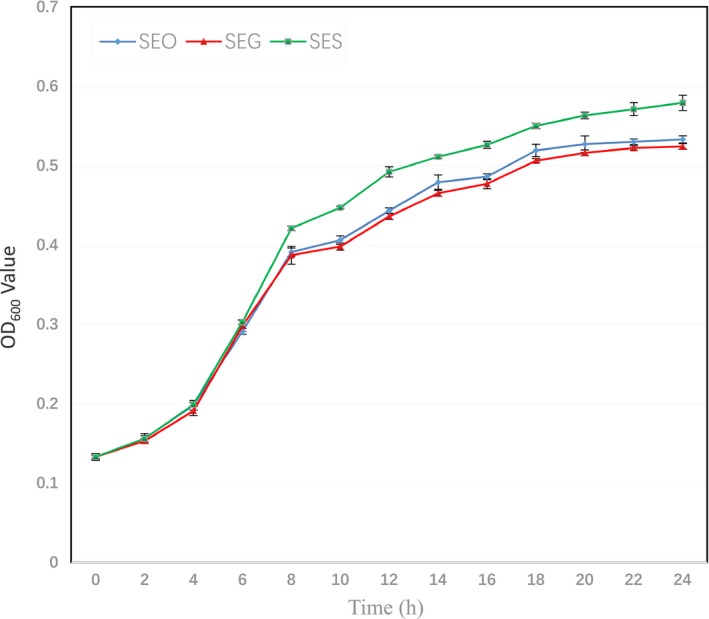
Growth curves of three *S. enteritidis* strains. Growth curves of SEO (blue), SEG (red), and SES (green) were determined by measuring the OD_600_ value, which represents the bacterial concentration. The OD_600_ value was measured every 2 hr for 24 hr

Growth rate data for the three strains are shown in Table 1. Compared with the ground strain SEG, the spaceflight strain SES exhibited an increased growth rate, especially after 8 hr (*p = *0.0034), while the original strain SEO showed a similar growth curve to SEG (Figure 1).

**Table 1:** OD_600_ value of three *S. enteritidis* strains

Time (hr)	SEO	SEG	SES
0	0.133 ± 0.002	0.133 ± 0.004	0.133 ± 0.004
2	0.156 ± 0.006	0.153 ± 0.003	0.156 ± 0.004
4	0.198 ± 0.006	0.191 ± 0.006	0.199 ± 0.002
6	0.298 ± 0.004	0.291 ± 0.007	0.303 ± 0.003
8	0.391 ± 0.005	0.387 ± 0.011	0.421 ± 0.003
10	0.406 ± 0.005	0.398 ± 0.005	0.447 ± 0.002
12	0.443 ± 0.004	0.436 ± 0.004	0.492 ± 0.007
14	0.479 ± 0.009	0.465 ± 0.004	0.511 ± 0.002
16	0.486 ± 0.004	0.477 ± 0.006	0.526 ± 0.005
18	0.519 ± 0.008	0.506 ± 0.003	0.550 ± 0.004
20	0.527 ± 0.010	0.516 ± 0.004	0.563 ± 0.004
22	0.530 ± 0.004	0.522 ± 0.003	0.571 ± 0.008
24	0.533 ± 0.005	0.524 ± 0.004	0.579 ± 0.010

**Figure 2:**
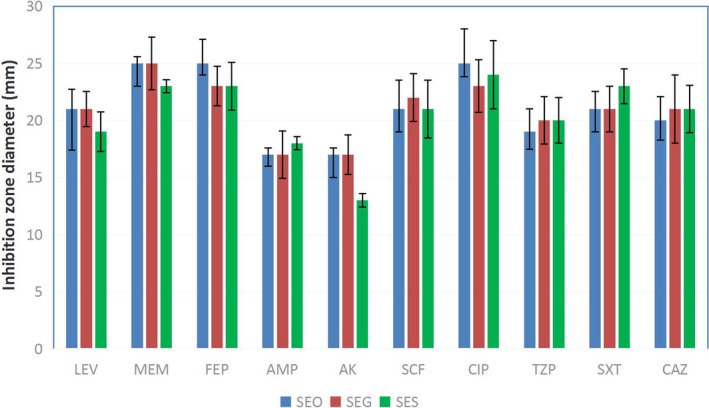
Antibiotic susceptibility test. The antibiotic susceptibility was determined by a disk diffusion test, and the inhibition zone diameter was measured for each antibiotic. According to the CLSI M100‐S24 document, the strain was considered resistant to AK if the inhibition zone diameter was not more than 14 mm after incubation of the bacterial plate. The data showed that SES (green) was resistant to AK, but SEO (blue) and SEG (red) were not

Antibiotic susceptibility tests indicated that all three strains exhibited similar inhibition zone diameters after incubating with nine antibiotics, including LEV, MEM, FEP, AMP, SCF, CIP, TZP, SXT and CAZ. However, the inhibition zone diameters of SEO, SEG, and SES to AK were 17 mm, 17 mm, and 13 mm, respectively. According to the CLSI M100‐S24 document (CLSI, 2014), SEO and SEG were susceptible to AK, while SES became resistant to AK (Figure 2).

To better understand the biochemical features of the three strains, 71 carbon source utilization and 23 chemical sensitivity assays were performed in this study. There were some differences in carbon source utilization and chemical sensitivity among the three strains (Table 2). Compared with SEO and SEG, SES showed decreased carbon source utilization and chemical sensitivity of pH 5 (A12), α‐d‐lactose (B2), α‐d‐glucose (C1), d‐sorbitol (D1), l‐glutamic acid (E6), β‐hydroxy‐d, l‐butyric acid (H4), and α‐ketobutyric acid (H5), indicating that the metabolism pathways of SES experienced some inhibited changes during spaceflight. In addition, the SEG and SES strains were shown to gain the ability to use the gelatin (E1) and vancomycin (F10) compared with SEO.

**Table 2:** Biochemical characteristics of three *S. enteritidis* strains

Substrate	SEO	SEG	SES
PH 5 (A12)	+	+	+ (w)
α‐d‐Lactose (B2)	+	+	+ (w)
α‐d‐Glucose (C1)	+	+	+ (w)
d‐Sorbitol (D1)	+	+	+ (w)
Gelatin (E1)	‐	+ (w)	+ (w)
l‐Glutamic acid (E6)	+	+	+ (w)
Vancomycin (F10)	‐	+	+
β‐Hydroxy‐d, l‐butyric acid (H4)	+	+	+ (w)
α‐Ketobutyric acid (H5)	+	+	+ (w)

Note

“+” represents a positive reaction; “‐” represents a negative reaction; “+ (w)” represents a weakly positive reaction.

### 3.2 Genome sequencing, assembly, and annotation

The accession number of SEO is CP033089‐CP033090. The SEO genome was assembled to a chromosome sequence and a plasmid sequence using the software Celera Assembler, which generated high‐quality genomic assemblies. The assembly statistics are shown in Table 3.

**Table 3:** The assembly data of SEO

Sequence type	Sequence topology	Sequence number	Total length (bp)	GC content (%)
Chromosome	Circular	1	4,676,321	52.17
Plasmid	Circular	1	59,373	51.95

**Figure 3:**
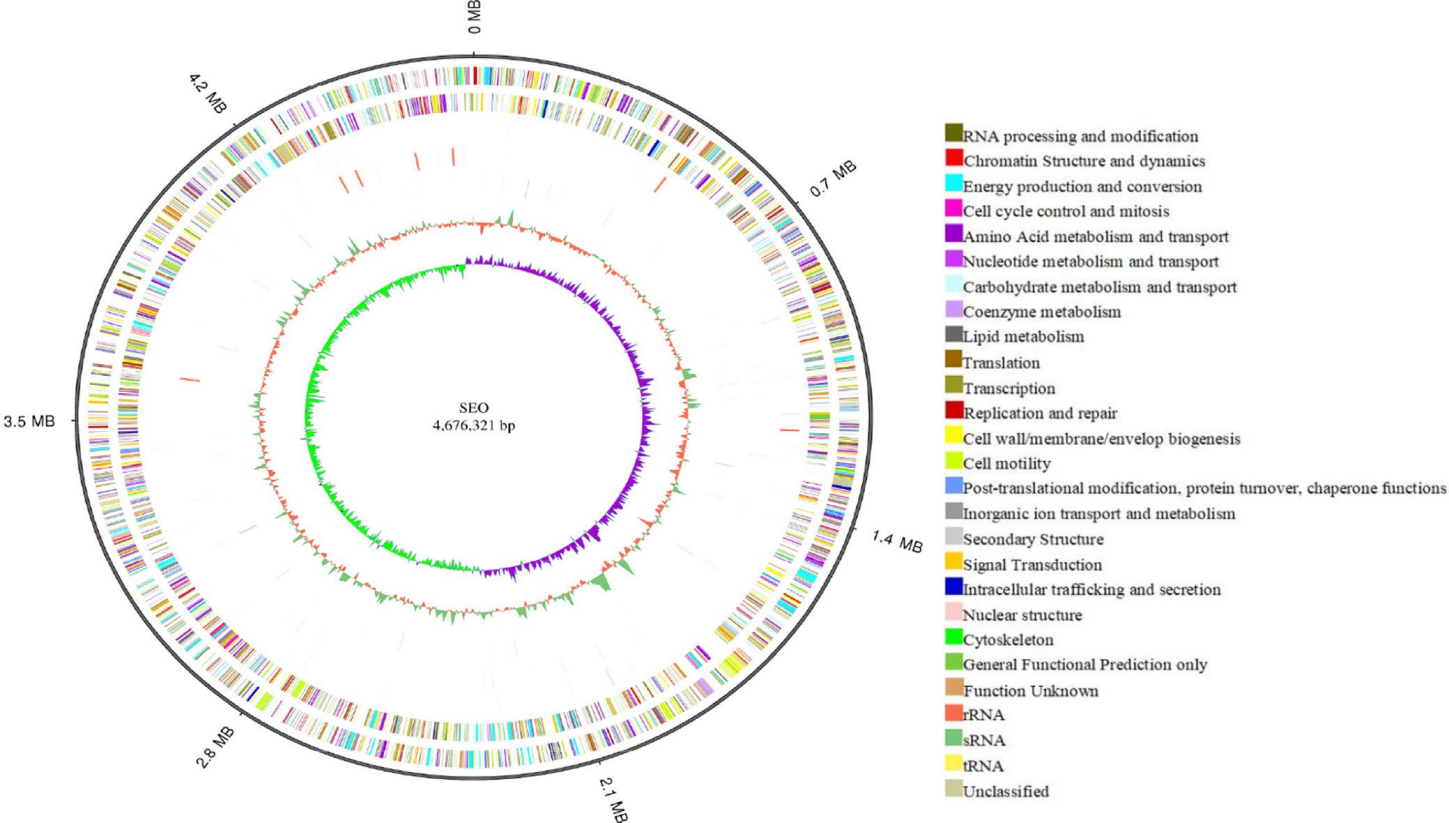
Genome map of the chromosome for SEO. From outer to inner, genome size (ring 1), COG annotation of positive‐strand genes (ring 2), COG annotation of negative‐strand genes (ring 3), positive‐strand ncRNAs (ring 4), negative‐strand ncRNAs (ring 5), repeat sequences (ring 6), GC content (ring 7), and GC skew (ring 8) are shown

**Figure 4:**
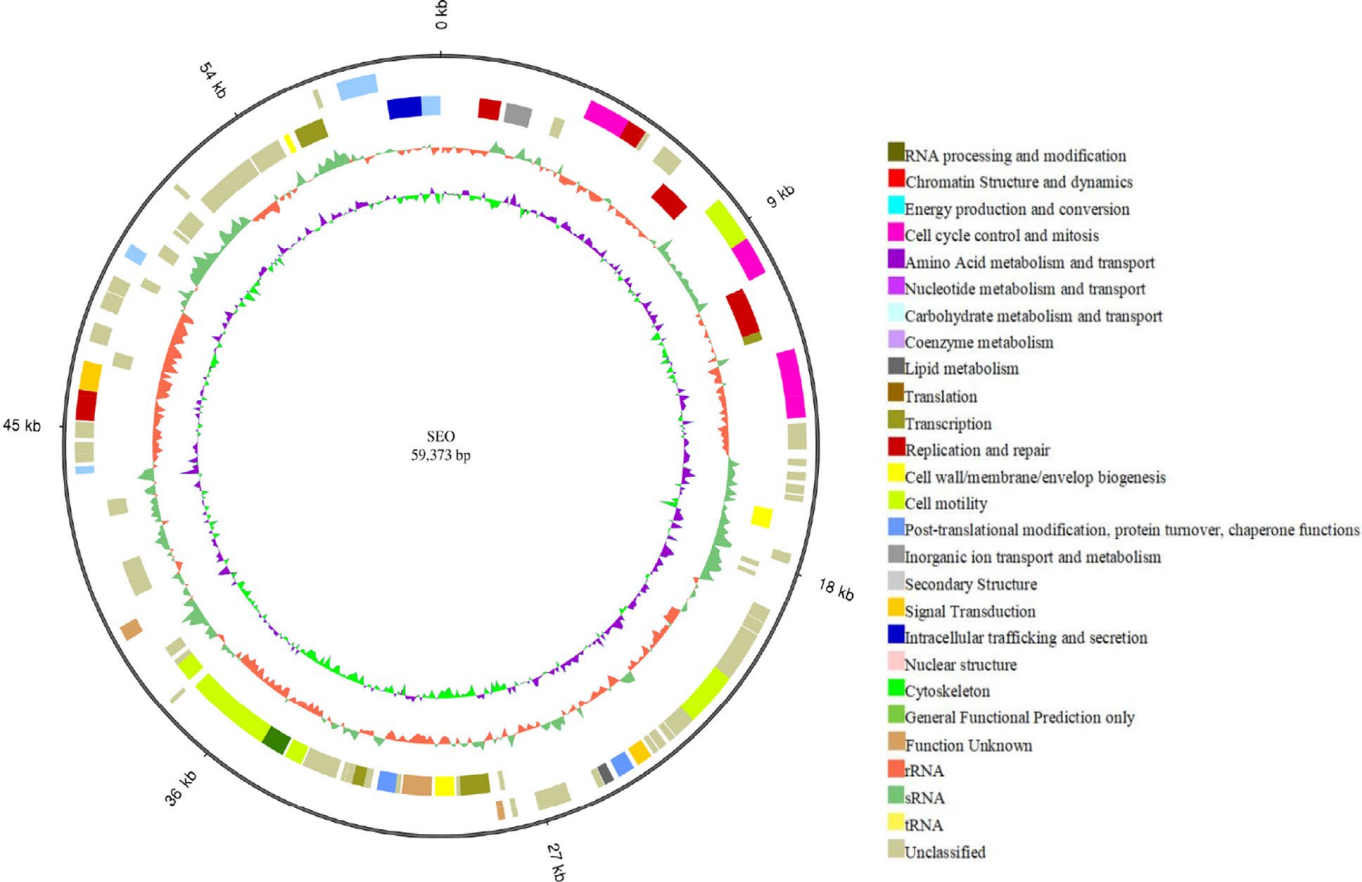
Genome map of the plasmid for SEO. From outer to inner, genome size (ring 1), COG annotation of positive‐strand genes (ring 2), COG annotation of negative‐strand genes (ring 3), GC content (ring 4), and GC skew (ring 5) are shown

The original strain SEO was used as a reference. Because all of the comparative genomic analyses were based on the data of the reference strain, the genome components and associated function of SEO were determined using different databases. The software Glimmer was used for gene prediction, and 4,636 genes with a total length of 4,108,686 bp that consisted of 86.76% of the genome were identified. Furthermore, 75 tandem repeat sequences with a total length of 11,043 bp, 51 minisatellite DNAs with a total length of 4,035 bp and five microsatellite DNAs with a total length of 211 bp were identified. In addition, 86 tRNAs, 68 sRNAs, 22 rRNAs, 6 prophages, and 2 CRISPRs were found in the chromosome. A circular map was drawn for each genomic sequence of chromosome (Figure 3) and plasmid (Figure 4).

**Figure 5:**
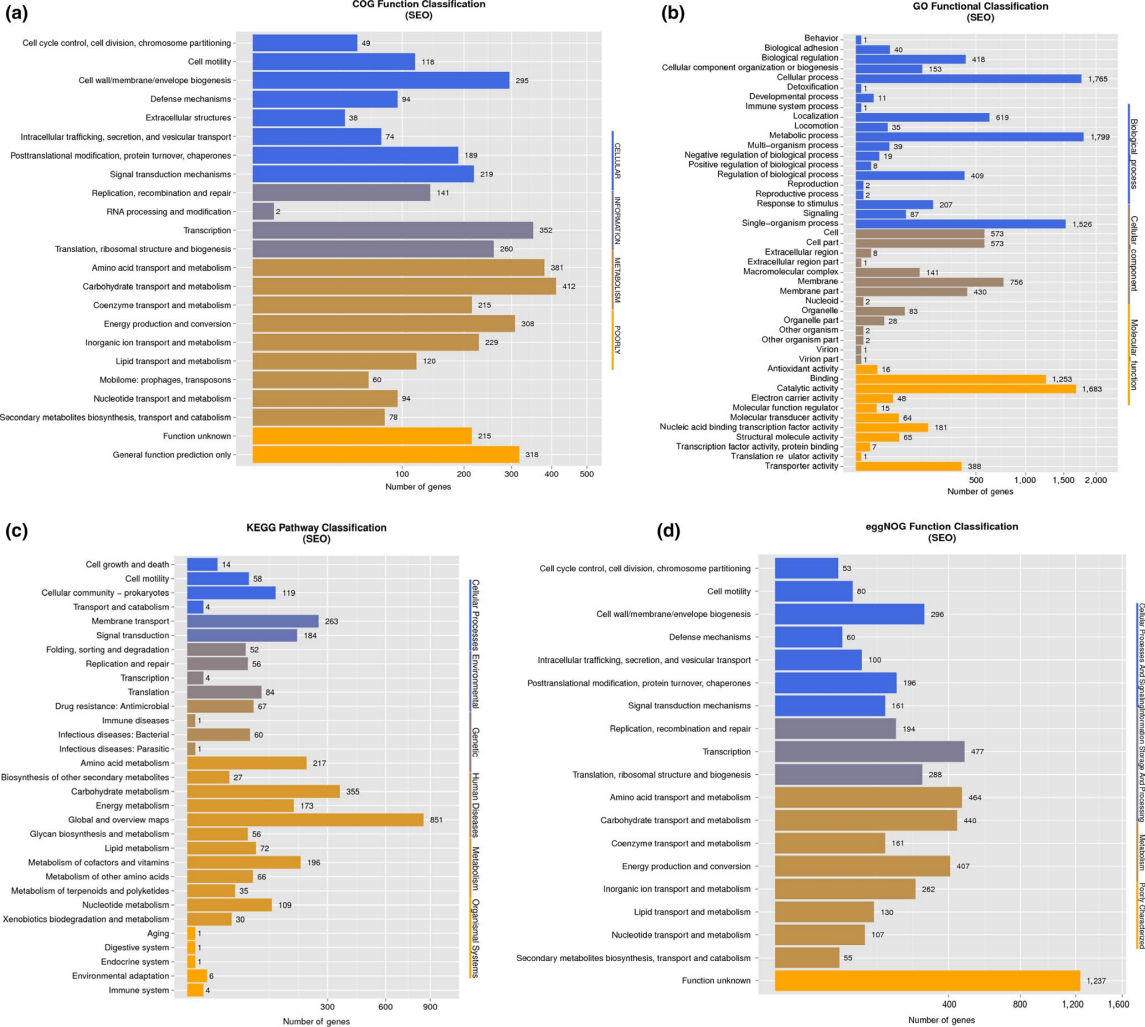
Function annotation of the genome of SEO. (a) COG function classification of the genome of SEO. (b) GO function classification of the genome of SEO. (c) KEGG pathway classification of genome of SEO. (d) eggNOG function classification of the genome of SEO

All the genes were annotated against the popular functional databases, including 78.94% of the genes using the COG database (Figure 5a), 66.97% of the genes using the GO database (Figure 5b), 69.37% of the genes using the KEGG database (Figure 5c), 29.09% of the genes using the eggNOG database (Figure 5d), 99.93% of the genes using the NR database, 77.35% genes using SwissProt, and 99.82% of the genes using TrEMBL.

### 3.3 Comparative genomics analysis

For SEG and SES, the accession numbers of resequencing data are SAMN10250723 and SAMN10251062, respectively. SEO is used as a reference strain and the whole genomic sequencing data of the SEG and SES strains are shown in Table 4. Genomic level comparisons, including SNP and indel calling, were performed among the three strains.

**Table 4:** The genomic feature of three *S. enteritidis* strains

Sample	SEO	SEG	SES
Genome size (bp)	4,735,694	4,690,190	4,689,311
Total number	4,636	4,609	4,610
Total length (bp)	4,108,686	4,077,888	4,078,278
Average length (bp)	886.26	884.77	884.66
Length/genome length (%)	86.76	86.95	86.97
GC content (%)	53.34	53.32	53.32

For SNP identification, compared with SEO, no SNP was found in the SEG and SES strains. To detect more variations, indels were analyzed using LASTZ software. Sixteen indels were found between the SEO and SEG strains, including seven indels in coding regions and nine indels in intergenic regions. Fourteen indels were detected between the SEO and SES strains, including five indels in coding regions and nine indels in intergenic regions. The details of the indels located in the CDS of the strains are shown in Table 5. One insert region identified in SEOGL003932 and one delete region identified in SEOGL002861 were located in the CDS of SEG, which were annotated as a transposase and an RNase Ⅲ regulator in the COG database, respectively.

**Table 5:** Summary of all indels located in the CDS of the SEG and SES strains

Strain	Indel type	Gene ID	Indel start	Indel end	Base	Annotation (NR database)
SEG and SES	Insert	SEOGL000217	97,339	97,340	A	Small toxic polypeptide ldrD
SEG and SES	Insert	SEOGL001389	145,155	145,156	C	Transposase
SEG and SES	Deletion	SEOGL001096	170,375	170,375	A	Transposase
SEG and SES	Deletion	SEOGL001389	145,192	145,192	CATGA	Transposase
SEG and SES	Deletion	SEOGL004159	683	683	G	Large repetitive protein
SEG	Insert	SEOGL003932	229,985	229,986	T	Transposase
SEG	Deletion	SEOGL002861	259,154	259,154	GACCTT	Tail assembly protein

### 3.4 RNA‐Seq alignment and comparative transcriptomic analysis

The accession number of transcriptomic data for SEG is SAMN10371527 and for SES is SAMN10371528. The sequencing reads of the SEG and SES strains were mapped to the reference genome of SEO (Table 6). The percentage of the total reads for the SEG and SES strains mapped to the reference strain were approximately 91.39% and 92.24%, respectively. The uniquely mapped reads for the SEG and SES strains were 85.27% and 86.39%, respectively.

**Table 6:** RNA sequencing data of the SEG and SES strains

Sample	SEG	SES
Total reads (bp)	21,855,835	21,851,158
Total base pairs (bp)	1,092,791,733	1,092,557,917
Total mapped reads (bp)	19,974,048 (91.39%)	20,156,237 (92.24%)
Total unmapped reads (bp)	1,881,059 (8.61%)	1,694,921 (7.76%)
Unique match (bp)	18,636,471 (85.27%)	18,877,944 (86.39%)
Multi‐position match (bp)	1,337,577 (6.12%)	1,278,293 (5.85%)

**Figure 6:**
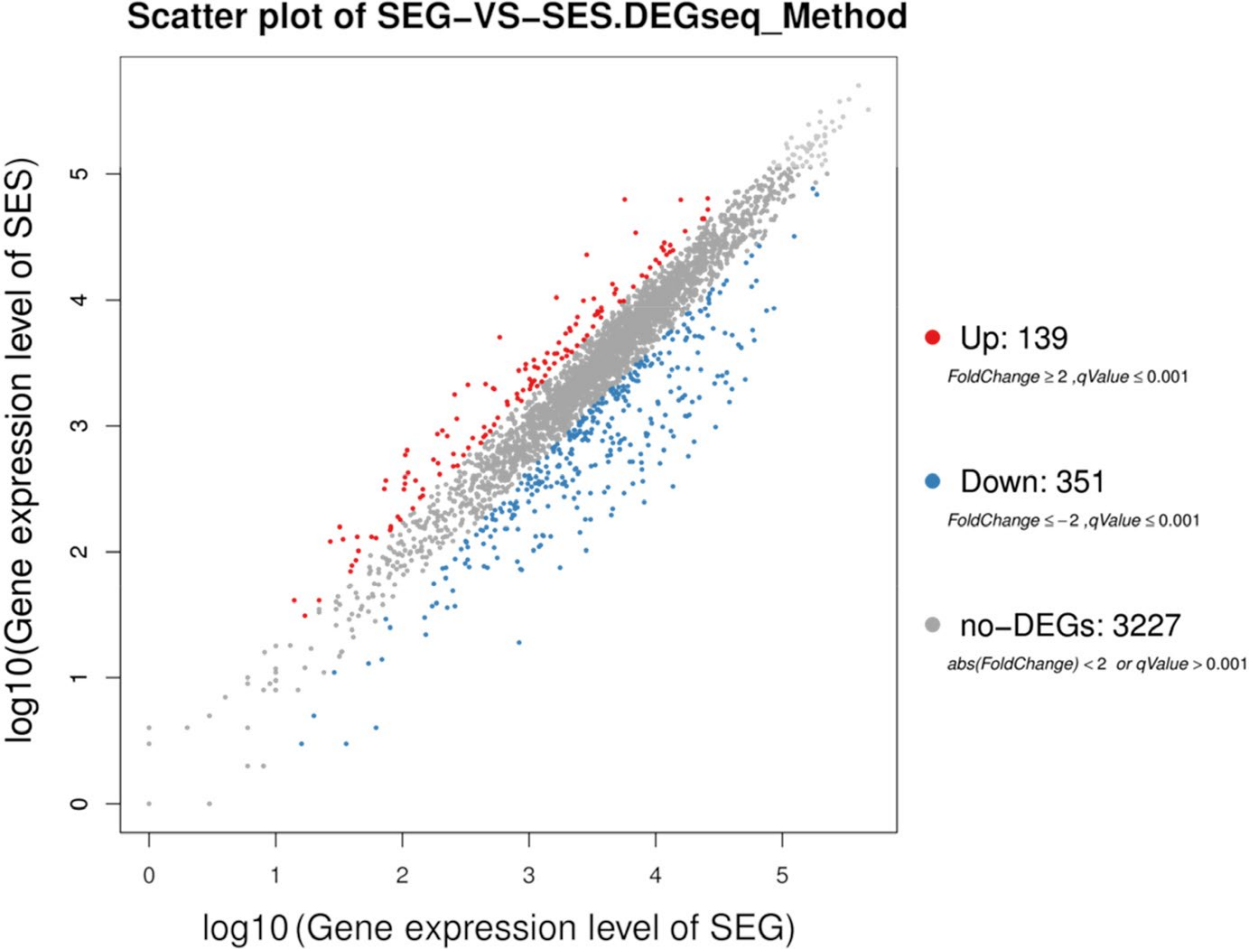
Global profiling of gene expression changes. The *X*‐axis represents the logarithm of the gene expression level for SEG, and the *Y*‐axis represents the logarithm of the gene expression level for SES. Red spots represent upregulated genes, blue spots represent downregulated genes, and gray spots represent the genes that did not change significantly

**Figure 7:**
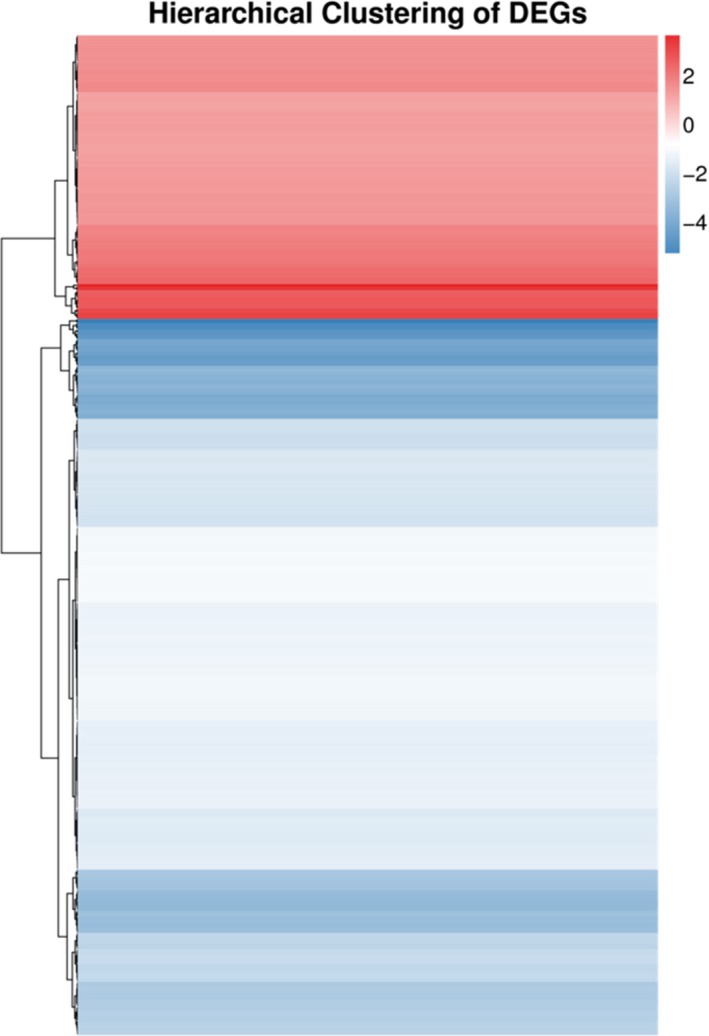
Hierarchical clustering analysis of DEGs for the SEG and SES strains. Blue bars represent the downregulated genes, red bars represent the upregulated genes, and white bars represent the genes that did not change significantly

A total of 490 DEGs were identified between the SEG and SES strains according to the FPKM values. Compared with SEG, SES contained 139 upregulated and 351 downregulated genes (Figure 6). Cluster analysis of DEG profiles is shown in Figure 7. The numbers of downregulated genes significantly exceeded those of upregulated genes, suggesting that gene expression and metabolism were inhibited in SES.

**Figure 8:**
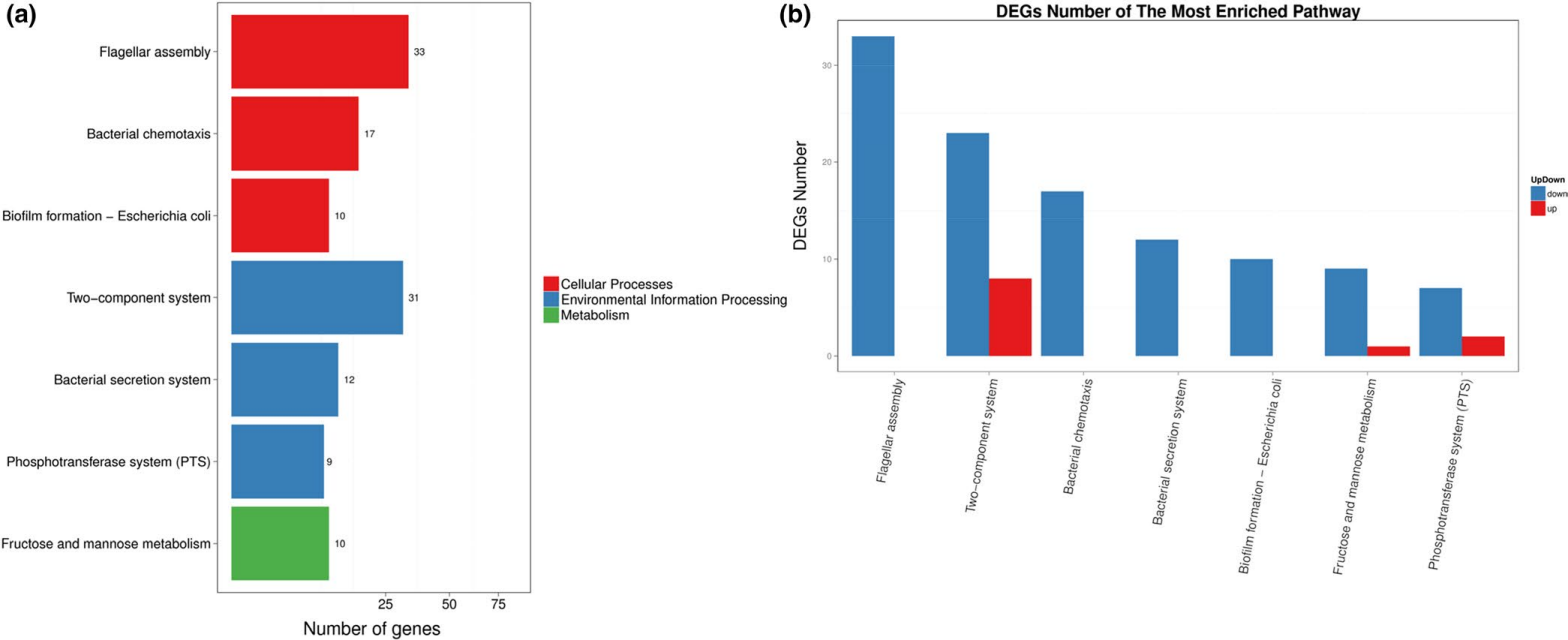
(a) KEGG pathway enrichment analysis of DEGs. The *X*‐axis represents the number of DEGs corresponding to the KEGG pathway. The *Y*‐axis represents the KEGG pathway. (b) KEGG pathway enrichment analysis of upregulated and downregulated DEGs. The *X*‐axis represents the KEGG pathway. The *Y*‐axis represents the number of DEGs corresponding to the KEGG pathways. Blue represents downregulated DEGs, and red represents upregulated DEGs

Differentially expressed genes were enriched and clustered to identify the metabolic pathways based on the KEGG pathway analysis. Seven categories including 122 DEGs were particularly meaningful between the SEG and SES strains. In comparison to SEG, the transcriptome of SES was mainly characterized by the regulation of genes involved in flagella assembly (*p = *6.49e‐29), two‐component system (*p = *0.00033819), bacterial chemotaxis (*p = *1.54e‐14), bacterial secretion system (*p = *0.00120393), fructose and mannose metabolism (*p = *0.007233108), biofilm formation (*p = *0.01687271), and phosphotransferase system (*p = *0.0329651) (Figure 8a). The upregulation and downregulation of DEGs were further analyzed according to the KEGG functional cluster. The genes associated with flagellar assembly, bacterial chemotaxis, bacterial secretion system, and biofilm formation were downregulated significantly, while the genes involved in phosphotransferase system, fructose and mannose metabolism, and two‐component system contained both upregulated and downregulated genes (Figure 8b). Notably, 10 DEGs including only one upregulated gene (*fucA*) and nine downregulated genes (*manX, manY, manZ, gutD, srlA, srlB, srlE, rfbK, *and* cpsB2*) were classified into the “Fructose and mannose metabolism” pathway. Additionally, nine DEGs including two upregulated genes (*STY2572 *and* treB*) and seven downregulated genes (*manX, manY, manZ, srlA, srlB, srlE, *and* ptsG*) were categorized into the “Phosphotransferase system (PTS)”pathway.

### 3.5 Comparative proteomic analysis

**Figure 9:**
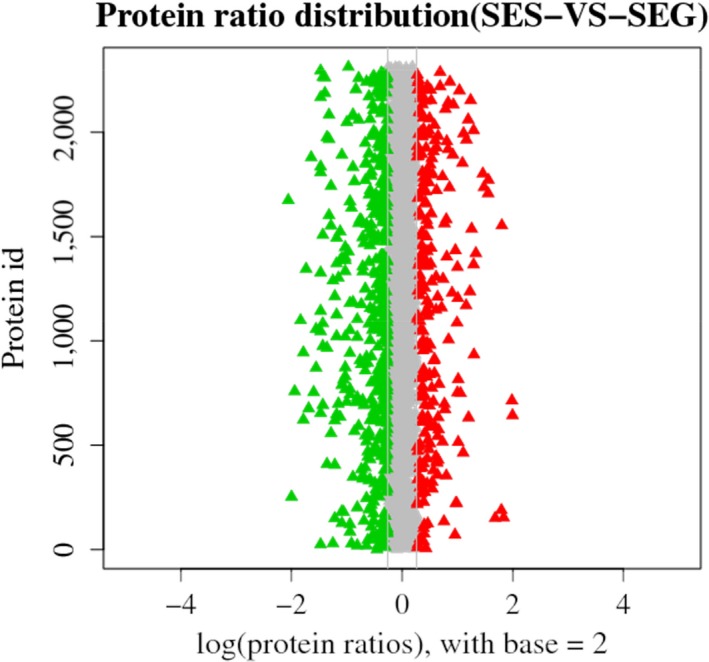
Protein ratio distribution between the SEG and SES strains. The distribution of the average protein quantification value is shown. Red triangles represent upregulated proteins, and green triangles represent downregulated proteins

**Figure 10:**
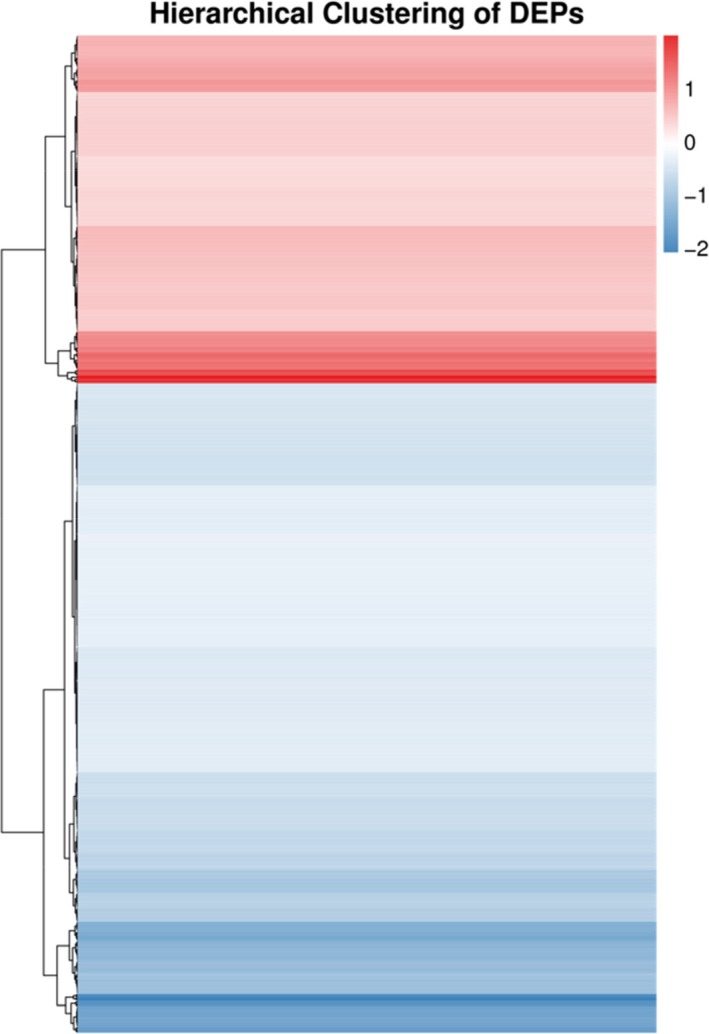
Hierarchical clustering analysis of DEPs. Blue bars represent the downregulated proteins, red bars represent the upregulated proteins, and white bars represent the proteins there did not change significantly

The accession number of proteomic data of SEG and SES is PXD011651. An analysis of the proteomic data revealed 653 DEPs between the SEG and SES strains, among which 425 proteins were downregulated and the remaining 228 proteins were overexpressed (Figure 9). The numbers of downregulated proteins significantly exceeded those of upregulated proteins, suggesting that protein expression and metabolism were inhibited in SES. The hierarchical clustering analysis of DEPs was performed using Euclidean distance and a hierarchical algorithm (Figure 10). Then, these DEPs were mapped to KEGG pathways and COG categories.

**Figure 11:**
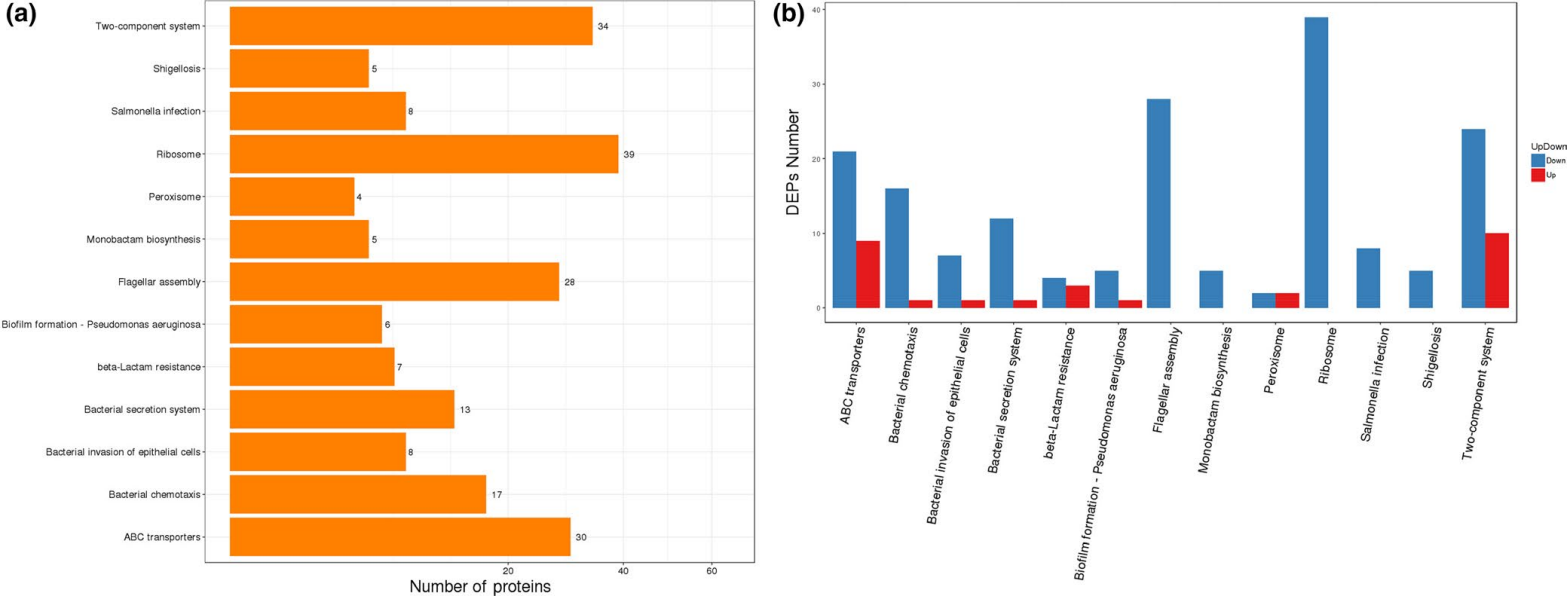
(a) KEGG pathway enrichment analysis of DEPs. The *X*‐axis represents the number of DEPs corresponding to the KEGG pathway. The *Y*‐axis represents the KEGG pathway. (b) KEGG pathway enrichment analysis of upregulated and downregulated DEPs. The *X*‐axis represents the KEGG pathway. The *Y*‐axis represents the number of DEPs corresponding to the KEGG pathways. Blue represents downregulated DEPs, and red represents upregulated DEPs

For KEGG pathway analysis, 13 KEGG pathways of 204 DEPs were statistically significant between the SEG and SES strains. It was obvious that the expression of proteins related to functions such as ribosome (*p = *1.45e‐12), flagellar assembly (*p = *4.23e‐17), two‐component system (*p = *0.01337264), and ABC transporters (*p = *0.01534541) changed the most (Figure 11a). Further analysis indicated that the DEPs related to flagellar assembly and ribosome were downregulated, while the DEPs involved in two‐component system and ABC transporters included both upregulated and downregulated proteins (Figure 11b), which was in accordance with the DEGs based on the KEGG pathways at the transcriptomic level.

**Figure 12:**
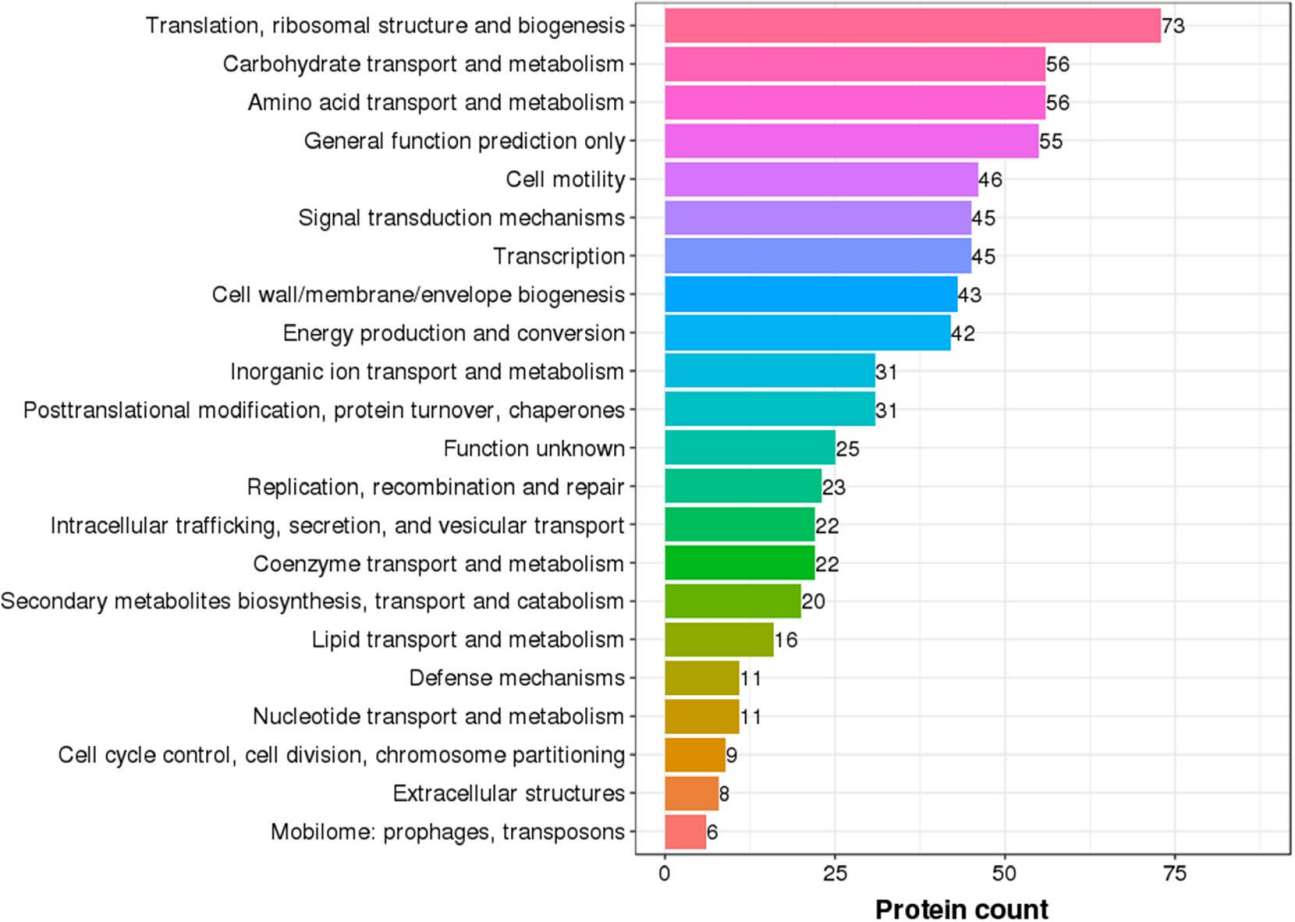
COG function classification of DEPs. The *X*‐axis represents the number of DEPs according to the COG function class. The *Y*‐axis represents the COG function class

Subsequently, DEPs were classified according to COG functional annotations. Twenty‐two categories, including 696 DEPs (the same proteins might fall into different categories), were shown to be meaningful (Figure 12). Among all the categories, translation, ribosomal structure, and biogenesis were the most significantly clustered items in DEPs, followed by amino acid transport and metabolism. Strikingly, 24 DEPs involved in cell wall/membrane/envelope biogenesis were upregulated, including some outer membrane proteins, lipoproteins, and mechanosensitive channel proteins. In addition, some overexpressed proteins, including cytochrome C‐type biogenesis protein, NADH dehydrogenase, and propionate kinase, were identified in the energy production and conversion category.

### 3.6 Integration of transcriptomic and proteomic analysis

To clarify the impacts of the space environment on *S. enteritidis* from a systematic biology perspective, the DEGs and DEPs were integrated to detect the overlapping genes expressed differently in both the transcriptome and proteome. Interestingly, the results revealed that the genes *oppA *and* oppD *were dramatically downregulated in both the transcriptomic and proteomic analyses. The *oppA *and* oppD *genes were described as part of many oligopeptide permease (*opp*) operons: the former gene played a role in coding oligopeptide ABC transporter substrate‐binding protein OppA, and the latter gene took part in the synthesis of oligopeptide ABC transporter ATP‐binding protein OppD.

## 4 DISCUSSION

Monitoring of the microorganisms on the space station is greatly important to evaluate risk factors for the health of astronauts and to assess material integrity of the spacecrafts (Liu, 2017). It has been reported that modeled reduced gravity and the space environment impacted microbial physiology, including growth rate, biofilm formation, antibiotic resistance, virulence, and metabolism (Byloos et al., 2017; Castro, Nelman‐Gonzalez, Nickerson, & Ott, 2011; Fajardo‐Cavazos, Narvel, & Nicholson, 2014; Horneck, Klaus, & Mancinelli, 2010; Orsini, Lewis, & Rice, 2017; Zea et al., 2018). The changes of microbes were adaptive responses to the microgravity environment, which allowed microbes to better accommodate the complicated environment. Spaceflight also modulated gene expression in the whole blood of astronauts, including those genes important for DNA repair, oxidative stress, and protein folding/degradation, which led to alterations in their physiology and potentially increased susceptibility to infectious diseases (Barrila et al., 2016). The host–microbe interactions were substantially affected under the human spaceflight environment and the analyses of microbial activities were necessary for predicting microbial behaviors and developing precautionary countermeasures during spaceflight (Senatore, Mastroleo, Leys, & Mauriello, 2018). Understanding the dynamics of microbial dispersal, survival, and proliferation in the crewed habitat in space might help to define better strategies to monitor and control microflora, finally benefiting crew health (Yamaguchi et al., 2014). In this study, compared with SEG, SES contained significant differences in growth rate, antibiotic susceptibility, and biochemical characteristics. Then, further studies including a combination of genomic, transcriptomic, and proteomic data were performed to understand the related mechanisms. These results showed that the space environment had profound influences on the biological characteristics of the *S. enteritidis* strain.

Previous studies of microbes exposed to modeled reduced gravity and space environments reported different growth rates because of the differences in experimental conditions, including culture conditions, medium and individual species. A majority of studies revealed that the microgravity environment increased the growth rate of microbes (Benoit & Klaus, 2007; Kim et al., 2013; Vukanti, Model, & Leff, 2012). However, some studies showed that no difference was found in the growth rate between space flight and ground control cultures (Coil et al., 2016; Fajardo‐Cavazos, Leehan, & Nicholson, 2018). Moreover, a few studies indicated that the flight strain performed significantly decelerated growth compared with the ground control experiment (Fajardo‐Cavazos & Nicholson, 2016). In this study, compared with SEG, SES exhibited an increased growth rate. The mechanism underlying the increased growth rate remains unclear, but it could be associated with enhanced utilization efficiency of the nutrients and reduced extracellular transport (Brown, Klaus, & Todd, 2002). According to KEGG pathway, the increased growth rate of SES might be associated with the DEPs involved in ABC transporters system. Moreover, based on the COG function analysis, some upregulated DEPs in the energy production and conversion category were identified in SES, which might play a critical role in the increased growth rate of SES. In addition, the cell membrane might provide the best protection of cell integrity under stress conditions (Su et al., 2014). According to the COG annotations, 24 proteins related to outer membrane protein synthesis were overexpressed in SES. As such, we speculated that this response together with changes in transmembrane transport and energy production and conversion, might increase the growth rate of the spaceflight strain.

Spaceflight could affect the drug resistance phenotype of bacteria and a profound drug resistance capacity of ISS microorganisms against environmental stresses was detected during these years (Mora et al., 2016; Singh et al., 2018; Urbaniak et al., 2018). It has been demonstrated that the increased antibiotic tolerance of *Escherichia coli* in human spaceflight environment might be attributed to the upregulation of genes related to oxidative stress response (Aunins et al., 2018). Another study from China also pointed out that spaceflight changed the antibiotic susceptibility of *Klebsiella pneumonia* and resulted in bacterial resistance to sulfamethoxazole, the mechanism was that the spaceflight strain of *K. pneumonia *contained an additional copy of the *sul1* gene, which encoded a substituted form of dihydrofolate synthetase and could not be inhibited by the drug (Guo et al., 2014). However, a latest study indicated that spaceflight could not induce a wide range of changes in the phenotypic response of *Bacillus subtilis* cells to antibiotic susceptibility, with the possible exception of enoxacin (Morrison, Fajardo‐Cavazos, & Nicholson, 2017)**. **In this study, drug resistance of the spaceflight strain SES was specifically analyzed and a greatly increased amikacin resistance of SES was found. Amikacin is an aminoglycoside antibiotic mostly used in the clinical treatment of infectious diseases. Reduced aminoglycoside uptake could confer clinical resistance in Enterobacteriaceae and other Gram‐negative pathogens. In recent years, bacterial resistance to aminoglycoside antibiotics has gradually increased. Related mechanisms of aminoglycoside resistance included ribosomal target modification, inactivating‐enzymes production, and drug‐uptake reduction (Garneau‐Tsodikova & Labby, 2016; Labby & Garneau‐Tsodikova, 2013; Ramirez & Tolmasky, 2010). The uptake of aminoglycoside antibiotics is a complex process, and the active transport system plays an important role in aminoglycoside antibiotic accumulation (Kashiwagi et al., 1992). As one of the active membrane transport proteins with a conserved ATPase domain, ABC transporters supply energy for the absorption of nutrients and the metabolism of drugs (Nicolás, Barcellos, Hess, & Hungria, 2007). OppA is an oligopeptide ABC transporter substrate‐binding protein that constitutes an oligopeptide permease transport system that is responsible for the transport of oligopeptides into cells (Wium, Botes, & Bellstedt, 2015). Aminoglycoside antibiotics were transferred into cells through the oligopeptide permease transport system, and the reduced expression of OppA was associated with aminoglycoside resistance (Acosta, Ferreira, Ferreira, & Costa, 2005). It has been reported that the oligopeptide transport system was important for the uptake of aminoglycoside antibiotics since a decrease in OppA was observed in approximately 40% of spontaneous aminoglycoside‐resistant mutant (Kashiwagi et al., 1998). Another study showed no decease in OppA expression among aminoglycoside‐sensitive *Escherichia coli* strains from patients (Acosta, Ferreira, Padilla, Ferreira, & Costa, 2000). Therefore, we speculated that spaceflight caused the downregulated expression of the *oppA* gene, leading to a diminished OppA protein content in SES and subsequently enhanced amikacin resistance.

In addition to a few differences in growth rate and antibiotic susceptibility, some metabolic changes including utilization of carbon sources between the SEG and SES strains were also detected. From the transcriptomic perspective, the spaceflight strain diverged from the non‐spaceflight strain in the expression of various genes, such as *srlA *and *ptsG.* The *srlA* gene of *E. coli *encodes enzymes that participate in converting d‐sorbitol to fructose 6‐phosphate (McEntee, 1977). The downregulated expression of *srlA* in SES might lead to a weakly positive reaction to d‐sorbitol (D1). The *ptsG* gene encodes the major glucose transporter IICB^Glc^, which plays an important role during glucose metabolism (Shin, Cho, Heu, & Ryu, 2003). Thus, we proposed that the downregulated expression of the *ptsG* gene in SES reduced the level of IICB^Glc^ and finally resulted in a weakly positive reaction to α‐d‐glucose (C1). Compared with SEG, the weakly positive reaction of SES to α‐d‐lactose (B2), l‐glutamic acid (E6), β‐hydroxy‐d, l‐butyric acid (H4), and α‐ketobutyric acid (H5) suggested that the SES strain during spaceflight might decrease its metabolism to accommodate the space environment.

Exposure to the space environment could lead to bacterial changes at the genomic level, including spot mutations and insertion and deletion mutations (Fajardo‐Cavazos et al., 2018; Li et al., 2014). It had been reported that the samples of yeast *Saccharomyces cerevisiae *after spaceflight on the Russian space station Mir performed total or large deletion mutations in the ribosomal protein L *(rpsL) *gene sequence (Fukuda et al., 2000). In this study, comparative genomic analysis indicated that five indels were identified in SES, but no SNP was found. These indels were located in regions annotated as small toxic polypeptide ldrD, transposase, and large repetitive protein in the NR database. Although the biological function was different, each indel played an important role in altering bacterial energy metabolism to accommodate the external stimulus during spaceflight. Further studies are required to evaluate the effect of the space environment on bacteria at the genomic level.

It has been demonstrated that the space environment altered the transcript levels of some genes in bacteria (Morrison & Nicholson, 2018). In this study, transcriptomic and proteomic analyses revealed that the differentially expressed genes and proteins were primarily located in pathways including flagella assembly, two‐component system, bacterial chemotaxis, and bacterial secretion system according to KEGG pathway functions. These alterations were mainly associated with metabolic processes and the biochemical response of SES grown in the space environment to a certain extent. There were notably more downregulated differentially expressed genes and proteins than upregulated differentially expressed genes in SES, suggesting that SES slowed down certain energy metabolism to better adapt to spaceflight.

## 5 CONCLUSION

This study was the first to demonstrate the phenotypic, genotypic, transcriptomic, and proteomic changes of *S. enteritidis *in response to the extreme environment of space. The results showed an increase in growth rate and amikacin resistance of *S. enteritidis *after spaceflight. Moreover, differentially expressed genes and proteins associated with phenotypic changes were observed in *S. enteritidis*. We found that the resistance to amikacin might be attributed to the downregulated gene *oppA* and oligopeptide transporter OppA, the role of which might be a rational target for novel antimicrobials and vaccines against pathogenic bacteria (Garmory & Titball, 2004; Ren et al., 2015). Although the potential for the development of novel antimicrobials that used the OppA system has yet to be realized, there is no doubt that this approach will have important significance in the future. In China, the experiments of microbial characteristics had often been conducted after samples were returned back to Earth because of the limitations imposed by the nature of such spaceflight experiments. To date, the high cost of loading microbiological samples onto spacecraft and the difficult setting limited China's microbial experiments in the space environment. As a result, only a small number of bacteria were carried into the space environment by the Shenzhou‐11 spacecraft. Encouragingly, China has prepared for building a space station by the early 2020s, and then microbial space studies will be performed in the space station, which will make the results more stable and reliable. Even so, the present study might serve as a basis for future studies regarding the complex mechanism by which bacteria adapt to the space environment. Moreover, the insights obtained from the study of drug resistance‐related transcriptomic and proteomic changes of the *S. enteritidis *strain in response to space environment could also facilitate the development of potential strategies to prevent and treat infection in astronauts.

## CONFLICT OF INTERESTS

None declared.

## AUTHORS CONTRIBUTION

C Liu designed and coordinated the project. B Zhang and P Bai performed laboratory experiments. X Zhao, Y Yu, X Zhang, and D Li performed the data analysis. B Zhang wrote the manuscript with assistance from all authors. All authors read and approved the final manuscript.

## 6 ETHICS STATEMENT

None required.

## Data Availability

Data have been uploaded to GenBank. The accession numbers of the complete genome of SEO strain are CP033089 and CP033090. Accession numbers for resequencing data of SEG and SES are SAMN10250723 and SAMN10251062, respectively. Accession numbers for transcriptomic data of SEG and SES are SAMN10371527 and SAMN10371528, respectively. Proteomic data are accessible in ProteomeXchange and accession number for proteomic data of SEG and SES is PXD011651.
